# Chromium(VI) Removal from Water by Lanthanum Hybrid Modified Activated Carbon Produced from Coconut Shells

**DOI:** 10.3390/nano12071067

**Published:** 2022-03-24

**Authors:** Athanasia K. Tolkou, Soultana Trikalioti, Olina Makrogianni, Maria Xanthopoulou, Eleni A. Deliyanni, Ioannis A. Katsoyiannis, George Z. Kyzas

**Affiliations:** 1 Laboratory of Chemical and Environmental Technology, Department of Chemistry, Aristotle University of Thessaloniki, 54124 Thessaloniki, Greece; soultrik@gmail.com (S.T.); mariaxanth@chem.auth.gr (M.X.); lenadj@chem.auth.gr (E.A.D.); katsogia@chem.auth.gr (I.A.K.); 2 Department of Chemistry, International Hellenic University, 65404 Kavala, Greece; olinamacro@gmail.com

**Keywords:** chromium removal, adsorption, activated carbon, coconut shell, water treatment, lanthanum

## Abstract

Cr(VI) is considered to be the most hazardous and toxic oxidation state of chromium and hence the development of effective removal technologies, able to provide water with Cr(VI) below the drinking water limits (US EPA 100 μg/L, European Commission 50 μg/L, which will be reduced to 25 by 2036) is a very important issue in water treatment. This study aimed at examining the performance of activated carbon produced from coconut shells, modified by lanthanum chloride, for Cr(VI) removal from waters. The structure of the formed material (COC-AC-La) was characterized by the application of BET, FTIR and SEM techniques. The effect of the adsorbent’s dosage, pH value, contact time, initial Cr(VI) concentration and water matrix was examined with respect to Cr(VI) removal. The results indicated that the maximum Cr(VI) removal was observed at pH 5; 4 h contact time and 0.2 g/L of adsorbent’s dosage was adequate to reduce Cr(VI) from 100 μg/L to below 25 μg/L. Freundlich isotherm and pseudo-second order kinetic models fitted the experimental data sufficiently. The maximum adsorption capacity achieved was 6.3 μg/g at pH 5. At this pH value, the removal percentage of Cr(VI) reached 95% for an initial Cr(VI) concertation of 30 μg/L. At pH 7 the corresponding efficiency was roughly 60%, resulting in residual Cr(VI) concentrations below the anticipated drinking water limit of 25 μg/L of total chromium, when the initial Cr(VI) concentration was 50 μg/L. Consecutive adsorption and regeneration studies were conducted using 0.01 M of NaOH as an eluent to evaluate the reusability of the adsorbents, Results showed 20% decrease of adsorption capacity after 5 regeneration cycles of operation.

## 1. Introduction

Water pollution is one of the major global problems affecting human health and the environment, affecting safe water availability and this problem is expected to be aggravated in the future by climate change, increase in population density and increased demand for agricultural products [1,2]. One of the most widespread and toxic pollutants is hexavalent chromium. Chromium is considered as one of the common heavy metals, occurring in water and groundwater as a result of natural and anthropogenic activities [3]. Chromium toxicity levels depend on its different oxidation state [4], i.e., trivalent chromium Cr(III) and hexavalent Cr(VI), in aqueous solutions, with the latter (Cr(VI)) being the most hazardous and toxic [5], whereas Cr(III) is considered to be nontoxic and an important element for the human body, mainly because it can endorse protein metabolism, decrease glucose concentration in the blood as well as reduce the possibility of atherosclerosis and heart attack [6]. On the contrary, Cr(VI), which is easily absorbed by the human body through digestion, respiratory, skin etc., is of serious concern due to its high toxicity and carcinogenicity [7]. For example, there is a high risk of serious health problems such as eye and skin irritation, renal problems, bronchial asthma, severe diarrhea, corrosion of skin, kidney dysfunction, destruction of DNA and RNA and probably lung carcinoma in humans etc. [8].

United States Environmental Protection Agency (EPA) has a drinking water standard of 100 μg/L for total chromium [9] and the respective World Health Organization’s (WHO) permissible limit is 50 μg/L [10]. European Commission decided, to reduce the value by 50% of WHO’s limit, i.e., to 25 μg/L [11] and the new limit will have to be complied by 2036. It is worth noting that Cr(VI) and Cr(III) are covered by the total chromium drinking water standard. However, a number of studies has revealed that almost all chromium present in natural waters is in the form of Cr(VI), since Cr(III) has a very low solubility and practically cannot exceed the concentration of 5 μg/L [12] and EPA’s regulation [9] assumes that a measurement of total chromium is 100% Cr(VI).

Several studies have reported increased concentrations of Cr(VI) in natural waters, mainly in groundwaters, worldwide such as in the USA, Mexico, India, Canada, Italy, Greece, etc. [12,13,14]. Chromium (VI) cannot be removed efficiently by conventional treatment technologies, such as coagulation with iron and aluminum salts and adsorption with iron oxides. The most usually applied technology for Cr(VI) removal is its redox assisted coagulation by ferrous salts, in which Cr(VI) is reduced to Cr(III) and Fe(II) is oxidized to Fe(III). Cr(III) then goes out for solution, either by precipitation as Cr(OH)_3_ or by adsorption of Cr(III) on the Fe(III) oxides which are formed by Fe(II) oxidation [15]. Other technologies that have been studied for Cr(VI) removal from water are ion exchange [16], biological treatment [17], membrane filtration [18], and adsorption [13,19,20,21].

Many of these conventional technologies have several drawbacks, such as expensive operating conditions, high energy consumption, need of highly trained personnel, release toxic by-products and production of excessive amounts of sludge [22]. On the other hand, adsorption has several advantages compared to other techniques, more importantly that is being cost effective and more efficient in removal of heavy metals from water, especially for treatment of waters with concentrations slightly higher than the legislative limits. Several adsorbents have been studied for the removal of Cr(VI) from waters [23], such as nickel oxide nanoparticles [24], chitosan grafted [25] and polypyrrole [26] graphene oxide nanocomposites, biochar [27] etc. In particular, adsorbents based on activated carbon are very promising in terms of performance [28], surface area, adsorption capacity and fast reaction rate [29]. However, commercially activated carbon is a costly material and producing activated carbons from cheap raw materials or by products is a major issue.

Therefore, activated carbons from different materials has been produced in several studies such as from bamboo, coconut husks, willow peat, wood, lignite, coal and petroleum pitch [30]. Recently, a variety of low-cost agro-industrial, bio-based materials [31] and domestic wastes materials [32], have been examined as adsorbents for Cr(VI) removal from aqueous solutions, including treated waste newspaper [33], apricot kernel [34], apricot stone and hazelnut shell [35], sugarcane bagasse [36,37], longan seed [38], sawdust [39], etc.

Pure activated carbon does not adsorb efficiently Cr(VI) [40] and has to be modified [41] by incorporation in the structure of functional groups that are efficient in binding Cr(VI), while the functional groups of AC are negatively charged and rather nonpolar causing a weak adsorption for Cr(VI). For example, to increase the efficiency of activated carbon, its advantages are combined with the properties of metals, by impregnation of metals in the structure of activated carbon [42]. In the present study, we used lanthanum (La) for the modification [43] of activated carbon. Lanthanum is a relatively low-cost element, the salts of which exhibit good adsorption capacity [44], presenting a very strong affinity towards anions like phosphate, fluoride and chromate [45]. Lanthanum, was used as an additive [46,47] to modify the surface of adsorbents and several lanthanum salts have been tested for Cr(VI) removal. The lanthanum forms used in these studies was LaPO_4_, La(NO_3_)_3_ and LaO_3_ which were difficult to separate from solution and were instable at acidic pH values.

Several studies using un modified activated carbon for Cr(VI) removal from water have been published [48,49,50]. Moreover, the conditions that the experiments were conducted, are not relevant for drinking water treatment, i.e., very high dosage of materials and very high initial Cr(VI) concentration. For example, at the paper by Ali Atieh et al. [48], the minimum concentration of Cr(VI) tested was 200 μg/L. At the paper of Chen et al. [48], the initial concentration of Cr(VI) was about 80 mg/L and the results showed that the material was working only at pH 3 and at higher pH values removal was negligible. The paper of Vo et al. [48], shows results from initial Cr(VI) concentration 5–200 mg/L. In this work, the investigation started from as low as 30 μg/L of Cr(VI) and the intention is to investigate the ability of the material to reduce it to below 25 μg/L, which is the newly imposed by the European Commission Cr(VI) concentration limit in drinking water. Therefore, this comparison shows that there is space for improvement. In the present study, activated carbon produced by coconut shells was further modified by using lanthanum chloride, applied to the removal of Cr(VI) from waters as a novel adsorbent and the applied conditions were designed to be relevant to Cr(VI) in natural water sources, i.e., starting from as low as 30 μg/L Cr(VI) initial concentration, with the further aim to reduce them to concentrations below 25 μg/L. To the best of our knowledge, until to date, there is not any study that has examined the use of such a modified material, i.e., the use of activated carbon from coconut shells modified with lanthanum chloride for the removal of Cr(VI).

The objective of the present study was to investigate the adsorption capacity of Cr(VI) through batch experiments and clarify the possible adsorption mechanisms related to adsorption of Cr(VI) on the prepared COC-AC-La. The structure and the morphology of the modified activated carbon was studied by the application of FTIR and SEM characterization techniques. Furthermore, the effect of pH, initial Cr(VI) concentration, adsorbent’s dose, contact time and different water dilutions, were also studied. Kinetic and isotherm models were also applied to describe the adsorption process.

## 2. Materials and Methods

### 2.1. Materials

All reagents used were of analytical grade. Cr(VI) stock solution of 10 mg/L was prepared by dissolving K_2_Cr_2_O_7_ ACS, ISO, Reag. Ph Eur (Merck; Darmstadt, Germany) in deionized water and stored for further use in the experiments. Then required concentrations of Cr(VI) were obtained by diluting the stock solution. LaCl_3_ heptahydrate 98% (Merck; Darmstadt, Germany) was used for the modification of activated carbon. pH of initial samples was adjusted by adding the required amount of 0.01–0.1M NaOH ACS reagent, ≥97.0%, pellets (Sigma-Aldrich; St. Louis, Missouri, USA) or 0.01–0.1M HCl 37% (Panreac; Cranbury, NJ, USA).

### 2.2. Synthesis of Modified Activated Carbon

Activated carbon was synthesized from coconut shells (COC-AC) and then modified with lanthanum. Briefly, coconut shells after being washed with distilled water to remove dust and other inorganic impurities, were dried overnight at 100 °C to reduce the moisture content. The dry material was grounded and sieved to be of uniform particle size (+0.45–0.15 mm). 20 g of dry coconut shells as precursor were impregnated with 250 mL of the activation agent (KOH 2M) at room temperature for 24 h. The mixing was filtrated and placed in a furnace. All treatments were carried out at a constant heating rate of 10 K/min and with nitrogen (99% pure) flow of 300 STP cm3/min, which was kept during heating and cooling (while the activation temperature was 600 °C for 2 h). After cooling, the solid pyrolysis residues were washed with milli-Q distilled water until constant pH (measured with a pH meter HP, model CRISON 602, Crison Instruments, SA, Barcelona, Spain). The resulting activated carbon was dried at 100 °C for 24 h in vacuum furnace. This final product of activated carbon was abbreviated as COC-AC. Then, 5 g of COC-AC were mixed with 25 mL metal solution (impregnation with 1.8 g LaCl_3_) for 1 h at 298 K (25 °C) and then sonicated for 2 h. The mixture was then rinsed with distilled water and subsequently dried all over night at 60 °C. The final sample obtained (COC-AC-La), was cooled in room temperature in order to use it in subsequent adsorption experiments.

The proximate analysis (Table 1) shows the strong relationship of some characteristics potential of biomass for energy generation. This applies to the ultimate analysis. The proximate test usually describes the certain defining characteristics of the sample with regards to the mass proportion of the moisture content MC, volatile matter VM, fixed carbon FC, and the ash content. The thermogravimetric analyzer (Model Pyris 1, Perkin Elmer; Waltham, MA, USA) was used to obtain the values of the aforementioned parameters following the ASTM E1131-08 methods. The ultimate analysis is an elemental analysis that discovers the samples’ basic constitution of carbon C, hydrogen H, nitrogen N, oxygen O, sulphur S, and volatiles. It was carried out by ASTM D3176-09 standard procedure using the (Model TruSpec Micro CHNS, Leco; St. Joseph, MI, USA).

### 2.3. Analytical Determinations

Cr(VI) residual concentration was measured photometrically by the application of the DPC photometric method [51], based on the reaction of Cr(VI) with diphenylcarbazide (DPC), following the addition of H_2_SO_4_ to acidify the sample. After waiting 15 min for the reaction to take place, chromium absorption was measured at 540 nm, using an ultraviolet-visible (UV-Vis) spectrophotometer (WTW Spectroflex 6100; Innotech Instrumentation Co., Ltd., Hong Kong, China) and the relative values were corresponded to the standard curve of Cr(VI) ions to determine the residual concentration. The accuracy in this method results from having a detection limit at 5 μg/L. Total chromium concentration was measured by atomic absorption spectroscopy coupled with graphite furnace (Varian Zeeman AA240Z with GTA 120; Hansen Way Palo Alto, CA, USA), which has a detection limit of 1 μg/L.

### 2.4. Characterization Techniques,

FT-Infrared Spectroscopy (FTIR) analysis of the prepared modified activated carbon, was recorded in the range of 4000−400 cm^−1^, to determine the functional groups present on the surface of COC-AC-La, in order to interpret the possible mechanism of Cr(VI) adsorption, using Perkin Elmer FT-IR/NIR spectrometer Frontier, New York, NY, USA. Scanning electron microscopy (SEM) was used in order to observe the morphology of respective products of activated carbon prepared from coconut shell by using Jeol JSM-6390 LV, Tokyo, Japan scanning electron microscope. The surface area was determined by the BET analysis software; nitrogen isotherms were measured using AS1Win (Quantachrome Instruments, Boynton Beach, FL, USA) at liquid N_2_ temperature (77 K). The samples were degassed at 150 °C in a vacuum system at 10^−4^ Torr before the analysis.

### 2.5. Adsorption Experiments

Adsorption experiments have been carried out by introducing a quantity of adsorbent in 15 mL falcon tubes containing Cr(VI) solution at specified initial concentrations and at a constant temperature. The mixture was agitated using a Trayster overhead shaker and Loopster rotator at a constant stirring speed (80 rpm). A number of experimental variables such as pH (5–8), Cr(VI) initial concentration (30–200 μg/L), adsorbent dose (0.1–0.5 g/L) and contact time (5–240 min for kinetics and 24 h for equilibrium), have been independently varied by keeping other parameters constant during the experiments. After adsorption, water samples were collected from the tubes, filtered through 0.45 μm pore size nylon filter and the concentrations of residual Cr(VI) and other relevant parameters were determined in the filtrate. The experiments are repeated thrice and the presented results are the average of these three measurements. It should be noted that the leaching percentage of lanthanum was only 3% as calculated; the latter was taken into account in all adsorption evaluation.

The percentage removal (%*R*) of hexavalent chromium (Cr(VI) was determined from the following equation Equation (1):

(1) $$R\;\left(\%\right)=\left(\frac{C_{0}-C_{f}}{C_{0}}\right)\times 100\%$$

where *C*_0_ represents the initial Cr(VI) concentration (μg/L), *C_f_* represents the final Cr(VI) concentration after the treatment (μg/L).

The adsorption capacity of adsorbent was estimated by the calculation of equilibrium amount in the solid surface of particle (*Q_e_*) (μg/g) from the following Equation (2):

(2) $$Q_{e}=\frac{\left(C_{0}-C_{e}\right)\times V}{m}$$

where *C_e_* represents the Cr(VI) concentration (μg/L) at equilibrium, *V* (L) is volume of solution, and *m* (g) is the mass of the adsorbent used.

#### 2.5.1. Equilibrium Experiments

For the isothermal experiments, a fixed amount of adsorbent sample (g) was added to 10 mL of Cr(VI) solution (30–200 μg/L) in 15-mL falcon tubes. The adsorption experimental results were fitted to the Langmuir and Freundlich isotherm models.

The Langmuir model correlates the solid phase adsorbate concentration (*Q_e_*) and the uptakes to the equilibrium liquid concentration and is expressed as Equation (3):

(3) $$Q_{e}=\frac{Q_{m}K_{L}C_{e}}{1+K_{L}C_{e}}$$

where *Q_m_* represents the theoretical monolayer/maximum adsorption capacity (μg/g), and *K_L_* is related to the energy of Cr(VI) adsorption (L/μg).

The Langmuir (1918) theory assumes that the adsorbent has a limited adsorption capacity (*Q_m_*) while the adsorbate forms a monolayer on the adsorbent surface and that there is a lack of interface between the adsorbed molecules.

The Freundlich model outlines the interrelation between Cr(VI) equilibrium concentration (μg/L) with the uptake capacities, *Q_e_* (μg/g) of adsorbent and is expressed as Equation (4):

(4) $$Q_{e}=K_{F}C_{e}^{1/n}$$

where *K_F_* is a constant related to adsorption capacity while 1/*n* is a constant related to the intensity of adsorption or surface heterogeneity, while value of 1/*n* = 0 is for heterogeneous phase; value of 1/*n* < 1 is for a normal Freundlich isotherm; and value of 1/*n* > 1 indicates a cooperative adsorption.

#### 2.5.2. Kinetics Experiments

The pseudo-first-order kinetics and pseudo-second-order kinetics of Cr(VI) metal ions adsorption were also investigated and the calculated kinetic adsorption values were further analyzed to estimate the sorption rates as well as to determine the suitable rate expressions characteristic of possible reaction mechanism. The pseudo-first-order and pseudo-second-order models employed for the data analysis are as shown below at Equations (5) and (6), respectively:

(5) $$Q_{t}=Q_{e}(1-e^{-k_{1}t})$$

(6) $$Q_{t}=\frac{k_{2}Q_{e}^{2}t}{1+k_{2}Q_{e}t}$$

where *Q_t_* and *Q_e_* represent the amount of Cr(VI) adsorbed (μg/g) at time *t* (min) and at equilibrium, respectively *k*_1_ represents the pseudo-first-order rate constant (L/min), *k*_2_ represents the pseudo-second-order rate constant adsorption (g/mg min), and t indicates the contact time (min).

## 3. Results and Discussion

### 3.1. Characterization of COC-AC-La

#### 3.1.1. Physical Properties

The physical properties of laboratory prepared COC-AC-La adsorbent, were summarized in Table 2. The BET surface area, total pore volume, and average pore diameter of COC-AC-La were measured to be 139 m^2^/g, 1.121 cm^3^/g and 160.8 Å, respectively, which show that COC-AC-La is practically efficient for adsorption. Also, the elemental analysis of the COC-AC-La showed that the content of lanthanum in the COC-AC-La was 4.36%.

#### 3.1.2. Scanning Electron Microscopy (SEM)

**Figure 1:**
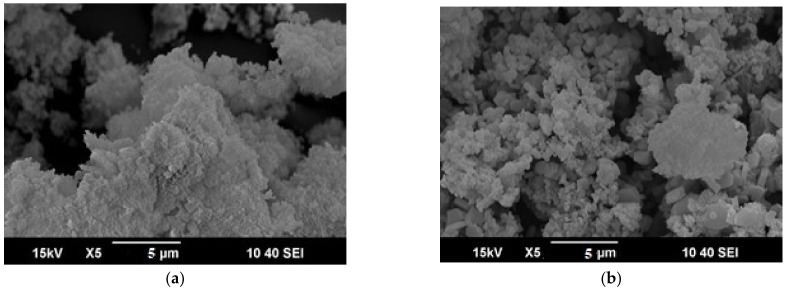
SEM images (**a**) COC-AC; (**b**) COC-AC-La.

The morphology and structure of the modified with lanthanum activated carbon prepared from coconut shells, were observed by SEM. As shown in Figure 1 COC-AC-La has a structure with pores and cavities that may be due to the carbonization stage during preparation [30]. In addition, asymmetric patterns of activated carbon pores have been detected that can create binding sites for different sizes of adsorbents. Also, it is observed that the surface of COC-AC was smoother before the doping with lanthanum, which is usually presented when an element is added to AC.

#### 3.1.3. FTIR Analysis

**Figure 2:**
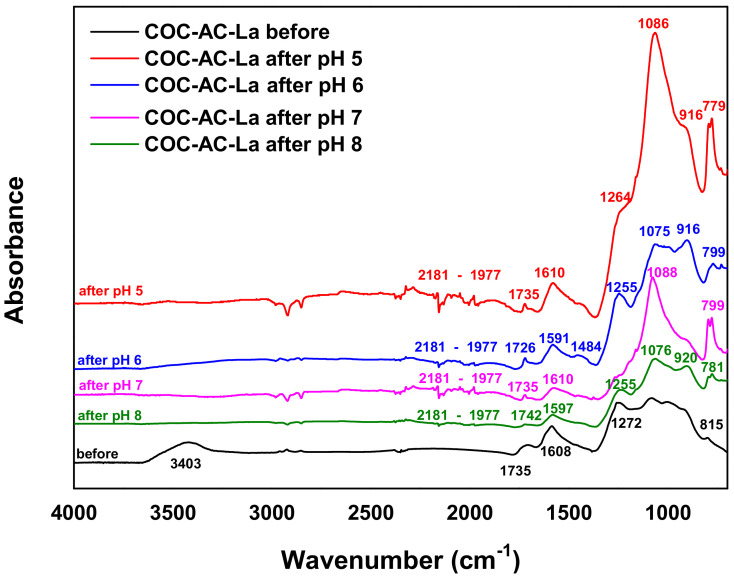
FTIR spectra of COC-AC-La before and after Cr(VI) adsorption at pH values 5, 6, 7 and 8.

The FTIR spectra of COC-AC-La before and after Cr(VI) adsorption at pH values 5 to 7 is illustrated in Figure 2. As depicted, in general the spectra are similar for all applied pH values, with an alteration in intensity and a formation of new bands, especially in comparison with COC-AC-La before adsorption of Cr(VI).

In particular, the first widest and stretched peak was obtained at 3403 cm^−1^ for the spectra of COC-AC-La before adsorption and corresponds to the symmetric and asymmetric O–H stretching vibration, which is in the range of 3200–3600 cm^−1^ and is characteristic for the presence of the hydroxyl group on the adsorbent material [52]. This hydroxyl group could be interrelated with organic acid, alcohol or phenol functional groups [43,52]. As observed, after adsorption of Cr(VI) on COC-AC-La, the peak of O–H at 3403 cm^−1^ disappeared and new peaks at around 1080 and 820 cm^−1^ were observed, most likely attributable to Cr–O–La bond [53,54], while this is an indicative peak for the stretching vibration of La = O groups. That means that especially the bonded -OH groups and carboxyl groups played a significant role in Cr(VI) adsorption on COC-AC-La. Furthermore, the peaks at around 2915, 2974, 2181–1977 and 1484 cm^−1^ on the FTIR spectra of COC-AC-La at all pH values after adsorption, were attributed to C–H, –C=C– (carbonyl) and C–C aromatic stretching, respectively [54]. In addition, the peaks at 1597/1610 cm^−1^ are associated with the C=C double bond. The peaks at 1255 cm^−1^ for pH 6 and 8 and 1264 cm^−1^ for pH 5 and at 1266 cm^−1^ for pH 7 are ascribed to C–O single bond [55,56]. Finally, the band in the range of 780–790 cm^−1^, which appeared only after the adsorption of Cr(VI), can be attributed to the formation of Cr–O bond [43,57]. It can be concluded that this comparative FTIR spectrum presented in Figure 2, clearly indicates the loading of Cr(VI) to the adsorbent [58].

### 3.2. Effect of Adsorbent Dose

**Figure 3:**
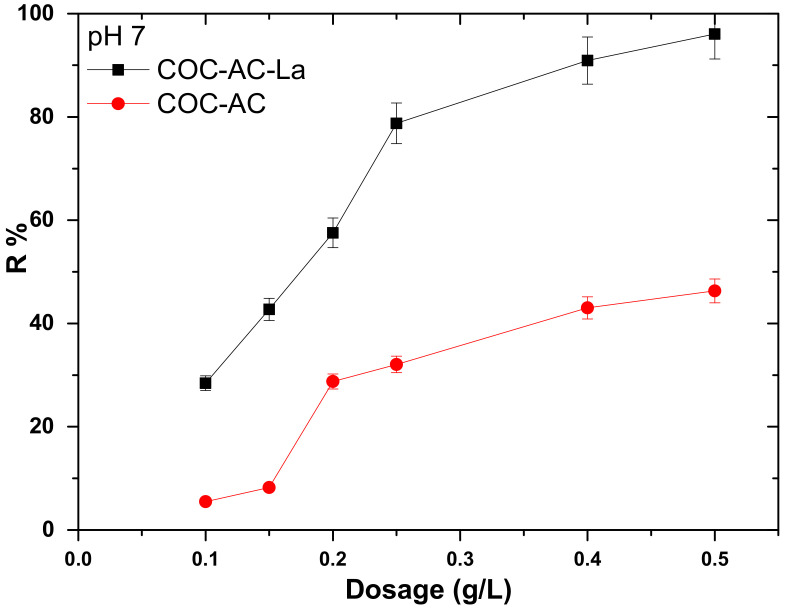
Effect of COC-AC-La dose on Cr(VI) adsorption, in comparison with COC-AC; initial Cr(VI) concentration 100 μg/L, pH 7.0 ± 0.1, T = 22 ± 1 °C, contact time 24 h, in deionized water.

In batch experiments, the dosage of the adsorbent was studied to determine the feasibility of the studied material for Cr(VI) removal. Experiments were conducted to study the effect of COC-AC-La dose on Cr(VI) adsorption in aqueous solution, by using an initial Cr(VI) concentration of 100 μg/L. Different concentrations of COC-AC-La were used: 0.10, 0.15, 0.20, 0.25, 0.4 and 0.50 g/L at neutral pH values, i.e., pH 7.0 ± 0.1, ambient temperature T = 22 ± 1 °C and contact time 24 h, in deionized water. Similar experiments were carried by using the unmodified (that means without lanthanum) activated carbon (COC-AC), in order to find out the differences in efficiency. The relevant results are quoted in Figure 3.

Figure 3 shows that with the increase of the adsorbent’s dosage, the percentage removal of Cr(VI) increased from 28% (using 0.10 g/L) to 96% (using 0.5 g/L). The latter may be due to the availability of more in number adsorption sites, as concluded from relevant studies [30,59]. Moreover, as it can be shown in Figure 3, when the dose is 0.25 g/L, the removal rate reaches 80%, with the residual Cr(VI) concentration being 21 μg/L, thus lower than the future limit of the European Commission, 25 μg/L [11]. The modification of the material showed advanced adsorption capacity, when comparing the results with those obtained by the use of the unmodified activated carbon. Cr(VI) removal by the COC-AC with 0.5 g/L resulted to only a 46% removal from 100 μg/L initial concentration of Cr(VI).

### 3.3. Effect of Initial Solution pH

To further understand Cr(VI) adsorption on La-modified activated carbon, the effect of pH was examined at pH range 5–8, these values are considered relevant for drinking water treatment, with constant adsorbent’s dose (0.2 g/L), initial Cr(VI) concentration of 100 μg/L, ambient temperature T = 22 ± 1 °C and contact time 24 h, in deionized water. The pH was controlled before the addition of the adsorbent with the required amount of 0.01–0.1 M NaOH or 0.01–0.1 M HCl.

**Figure 4:**
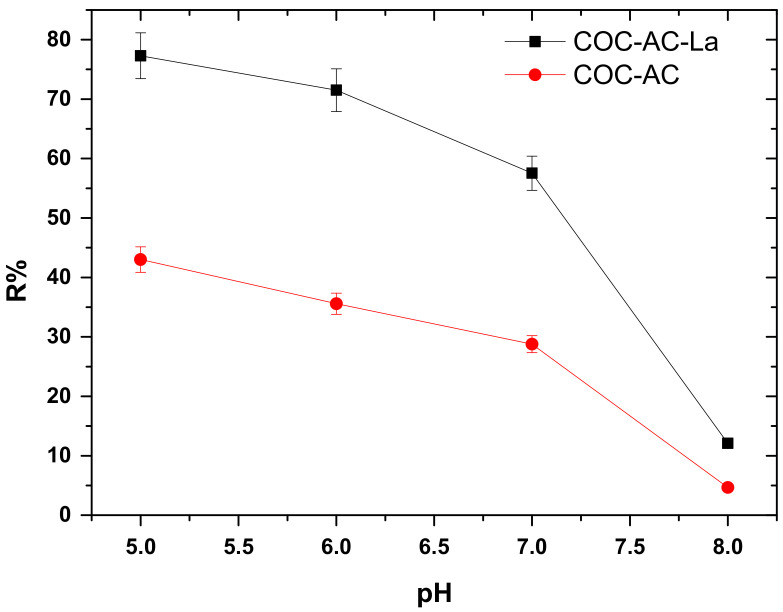
Effect of initial solution pH on the adsorption of Cr(VI) on COC-AC-La, in comparison with COC-AC; initial Cr(VI) concentration 100 μg/L, dose 0.2 g/L, T = 22 ± 1 °C, contact time 24 h, in deionized water.

As shown in Figure 4, the removal of Cr(VI) is favored at low pH values (mild acidic conditions) for both adsorbents. In particular, the highest removal percentage of Cr(VI) was achieved at pH 5 78% for the modified COC-AC-La and 44% for the unmodified carbon (COC-AC). Further increment of the pH, gradually resulted in reduced adsorbent capacity of COC-AC-La and at pH 8 there was a sharp decrease of the percentage removal of Cr(VI) which accounted only for 15%. Most likely, the point of zero charge (the pH values in which the surface charge of the material is neutral) of the modified material is found to be at pH 5 (approximately). At higher pH values, the overall surface charge of the material becomes negative, and as the pH value further increases, the overall negative value also increases. This hinders the binding of the negatively charged Cr(VI) species on the surface of the modified material, because of the increase of the repulsive forces between the negatively charged Cr(VI) species and the negatively charged surface. Cr(VI) in aqueous solutions is present as hydrogen chromate (HCrO_4_^−^), chromate (CrO_4_^2−^) and dichromate (Cr_2_O_7_^2−^), all anionic over the entire pH range [43,60,61].

**Figure 5:**
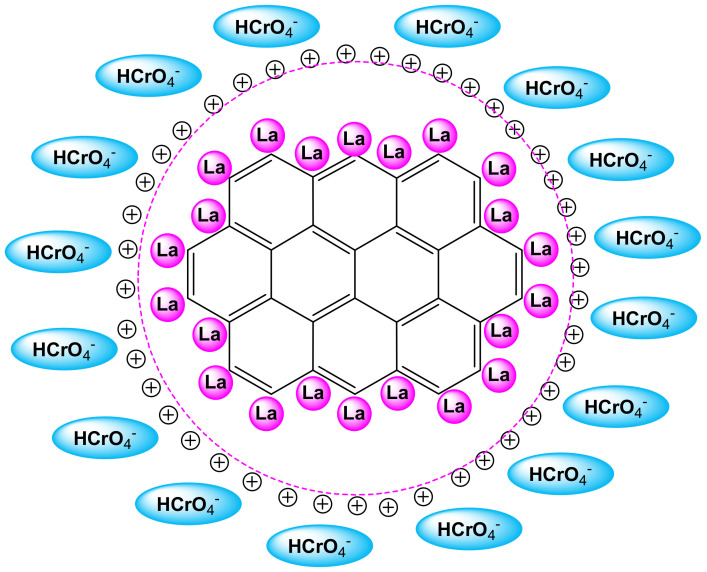
Possible interactions between COC-AC-La and Cr(VI).

It is worth noting that under the conditions of the experimental runs, it is very unlikely that Cr(VI) reduction could take place and Cr(VI) removal is taking place only through adsorption. Concurrently, the surface of COC-AC-La is positively charged, due to the deposition of lanthanum on the surface of activated carbon; and thus these predominant negative ions, HCrO_4_^−^ and Cr_2_O_7_^2−^, under acidic conditions, may attach to the positive surface functional groups of the adsorbent via electrostatic interactions [20,61,62,63]. This can also be confirmed by the difference in the adsorption capacity obtained in the case of the unmodified activated carbon. Figure 5 illustrates a possible interaction between COC-AC-La and Cr(VI) that might takes place.

### 3.4. Effect of Cr(VI) Initial Concentration

**Figure 6:**
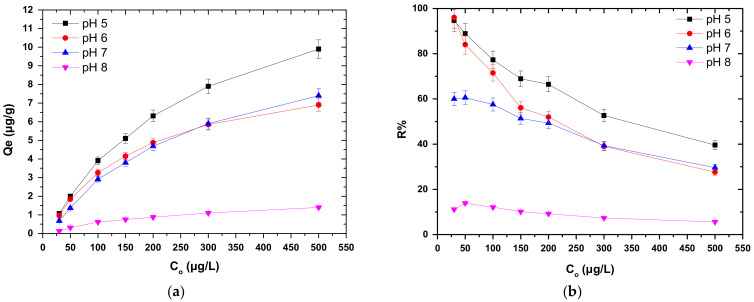
Effect of initial Cr(VI) concentration on the adsorption of Cr(VI) on COC-AC-La; in terms (**a**) of *Q_e_* and (**b**) of *R*% removal; initial Cr(VI) concentration 30–500 μg/L, solution pH 5–8, dose 0.2 g/L, T = 22 ± 1 °C, contact time 24 h, in deionized water.

The effect of Cr(VI) initial concentration was studied in the range of 30 to 500 μg/L, for 0.2 g/L dose of COC-AC-La adsorbent, at pH range 5 to 8, ambient temperature T = 22 ± 1 °C and contact time 24 h (Figure 5). The adsorption capacity of COC-AC-La (Figure 6a) was found to increase at pH value 5, 6, 7 and 8, from 1.1 to 6.3 μg/g, 1.0 to 4.9 μg/g, 0.7 to 4.7 μg/g and 0.13 to 0.86 μg/g, respectively. The increase in adsorption capacity with the increase of the initial Cr(VI) concentration may be attributed to the strong driving force of Cr(VI), which endorsed the surface adsorption of ions from the solution [63,64]. In addition, Figure 6 depicts a striking variation in adsorption efficiency of Cr(VI) on COC-AC-La between acidic and alkaline pH values. In relation to the removal rate (Figure 6b), at pH 5, the removal percentage of Cr(VI) is higher for all initial concentrations examined, thus considering it an effective adsorbent material for Cr(VI) removal.

### 3.5. Effect of Contact Time

**Figure 7:**
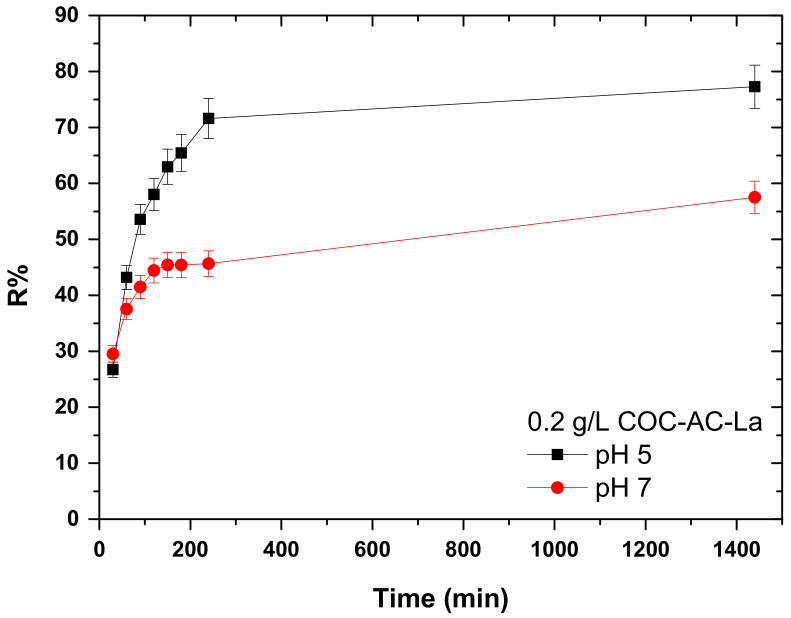
Effect of contact time on the adsorption of Cr(VI) on COC-AC-La; initial Cr(VI) concentration 100 μg/L, solution pH 5 and 7, dose 0.2 g/L, T = 22 ± 1 °C, in deionized water.

Another key-factor in adsorption process is the kinetic behavior of the adsorbent materials. In the present work, the effect of contact time from 30 to 240 min was studied by keeping all other parameters constant (initial Cr(VI) concentration 100 μg/L, solution pH 5 and 7, dose 0.2 g/L, T = 22 ± 1 °C) and the results are presented in Figure 7. The results showed that the percentage removal of Cr(VI) increased with the increase of contact time and kept constant after 120 min at pH 7 (55%). On the other hand, at pH 5 even after 240 min (72% removal) the removal continues slightly to increase (Figure 7). In Figure 4, it is shown that after 24 h, the removal of Cr(VI) at pH 5 accounts for 78%. Therefore, it can be considered that at 240 min, the major part of adsorption is completed. At pH 5, because of the abundance of positively charged species on the surface of the material, the adsorption continues further to proceed. At pH 7, because of the less prevalent negatively charged surface groups, the material gets quite fast saturated and the adsorption is almost completed after 90 min, though much less efficient [65]. In conclusion, so as to increase the cost-effectiveness of adsorption, 4 h (240 min) of contact time were selected as optimal time for batch experiments. This can be also confirmed from recent literature [66,67], as 240 min for Cr(VI) adsorption was also selected as optimum in acidic conditions.

### 3.6. Adsorption Isotherms

In order to study the mechanism of adsorption and define the association between the concentration of Cr(VI) and the adsorption capacity of the adsorbent, the adsorption isotherms experiments were conducted for initial concentrations of Cr(VI) in the range of 30 to 500 μg/L, for 0.2 g/L, adsorbent dose, at pH range 5 to 8, ambient temperature T = 22 ± 1 °C and contact time 24 h, in deionized water. The equilibrium adsorption of Cr(VI) ions on COC-AC-La was analyzed using adsorption isotherms models and is discussed in following.

#### 3.6.1. Freundlich Isotherm

**Figure 8:**
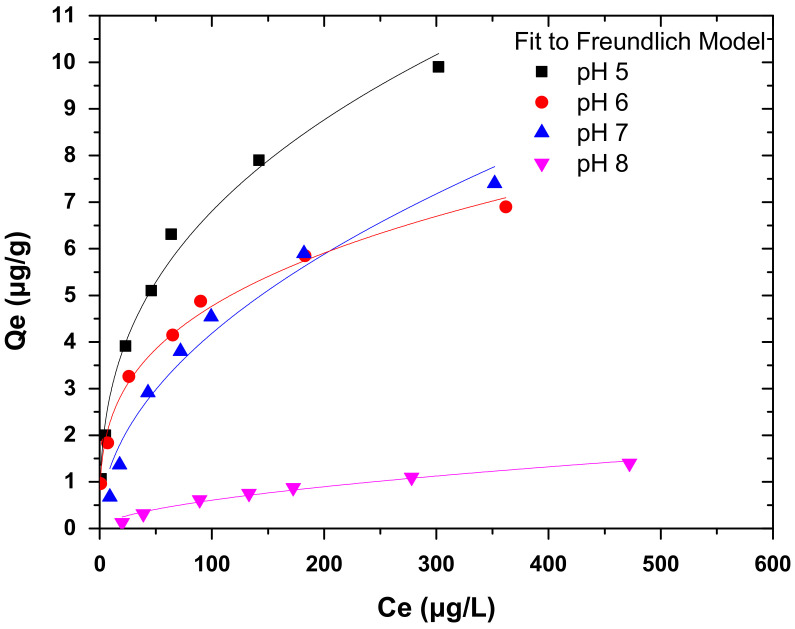
Freundlich isotherms for the adsorption of Cr(VI) on COC-AC-La; initial Cr(VI) concentration 30–500 μg/L, solution pH 5–8, adsorbent’s dose 0.2 g/L, T = 22 ± 1 °C, contact time 24 h, in deionized water.

The Freundlich isotherm model is applied in the case of multilayer adsorption. This model assumes the existence of interactions between adsorbed molecules. Figure 8 presents the Freundlich isotherm of Cr(VI) adsorption onto COC-AC-La at different pH values and the relative fitting parameters (calculated from Equation (4)) are presented in Table 2. As depicted, the correlation coefficient (R^2^) values are around 0.99 for pH 5, 6, and 7, and 0.97 for pH 8, and this indicates that the data of present adsorption studies fitted well to the Freundlich isotherm model, for all applied pH values.

Based on the graph, the values of *K_F_* and 1/*n* were calculated from the intercept and slope of the plots and for pH 5 the relative values were 1.27 and 0.36 for pH 6 were 1.15 and 0.31, for pH 7 were 0.44 and 0.49 and for pH 8 were only 0.05 and 0.56, respectively. For values of 1/*n* between 0 < 1/*n* <1, adsorption is favorable. The 1/*n* value of 0.36 and 0.31 for Cr(VI) in acidic conditions (i.e., pH 5 and 6, respectively) suggests that the adsorbent is effective for Cr(VI) adsorption and the adsorption process can be characterized as favorable and heterogeneous as well as chemisorption. In addition, the higher the values of *K_F_*, the better the adsorption performance is [30]. Hence, from the calculated parameters presented in Table 3, it is obvious that the adsorption of Cr(VI) in alkaline solutions (*K_F_* = 0.02 μg/g and 1/*n* = 0.73) is not effective, as also shown earlier in Figure 4 and Figure 5 and from the relative literature [30,43,60,61].

#### 3.6.2. Langmuir Isotherm

The results did not fit well, according to the Langmuir model (as R^2^ < 0.9), and thus they are not included in Table 3, whereas they were best fitted according to Freundlich model (as R^2^ > 0.973) as described above.

### 3.7. Adsorption Kinetics

In this study, two kinetic models were used to describe the adsorption of Cr(VI) on COC-AC-La; pseudo-first order model and pseudo-second order model. However, the pseudo-first-order model was initially applied in this study, but it did not correlate well to the obtained results (data not presented). Therefore, the experimental data were described with the pseudo-second order model, from which a better correlation was obtained.

#### Pseudo-Second Order Model

**Figure 9:**
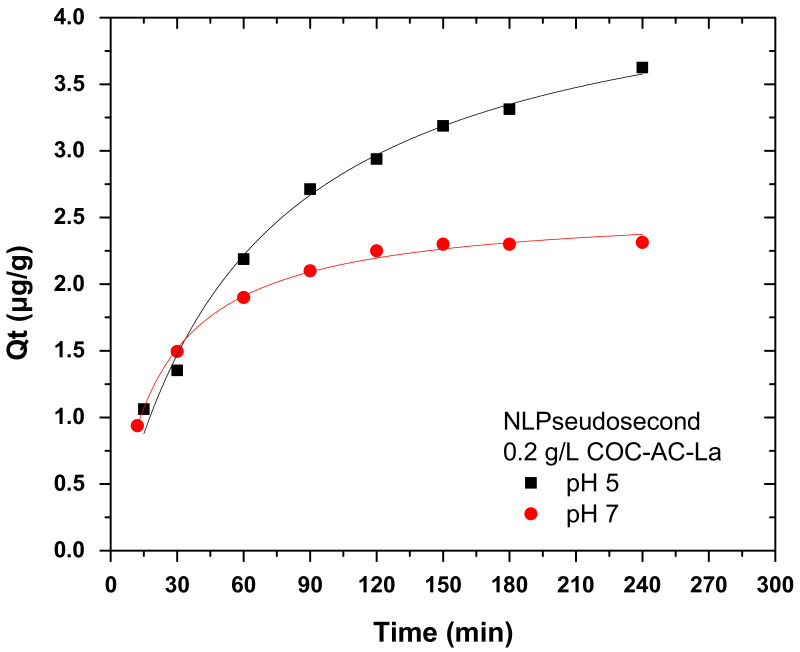
Kinetic pseudo-second order model for the adsorption of Cr(VI) on COC-AC-La; initial Cr(VI) concentration 100 μg/L, solution pH 5 and 7, dose 0.2 g/L, T = 22 ± 1 °C.

Figure 9 presents the pseudo-second order kinetic model for the adsorption of Cr(VI) on COC-AC-La with initial Cr(VI) concentration of 100 μg/L, at two different pH values, i.e., 5 and 7, by applying 0.2 g/L of the adsorbent at room temperature.

The pseudo-second order model constants at different pH values (calculated from Equation (6)) are given in Table 4. As shown, the R^2^ for both pH is very close to 1, indicating that the adsorption fitted excellent this model. The fitting results led to the conclusion that the adsorption of Cr(VI) on COC-AC-La was closer to chemisorption. The mechanism that carried out was an exchanging or sharing of electrons between the adsorbate and the adsorbent [30,61,68]. The calculated *Q_e_* values (*Q_e,cal_*) from the model, given in Table 4, relatively agree on the comparative experimental *Q_e_* (*Q_e,exp_*) values, confirming the above assumptions.

### 3.8. Effect of Water Matrix

To further investigate how the water matrix affects the removal efficiency of Cr(VI), tests in tap water as well as diluted solutions in different molar ratios of tap and deionized water, namely 1/1, 1/5, and 1/10 were carried out. The main physicochemical properties of the applied water types are presented in Table 5. It is worth noted that the tap water used for the experiments originates from groundwater sources.

**Figure 10:**
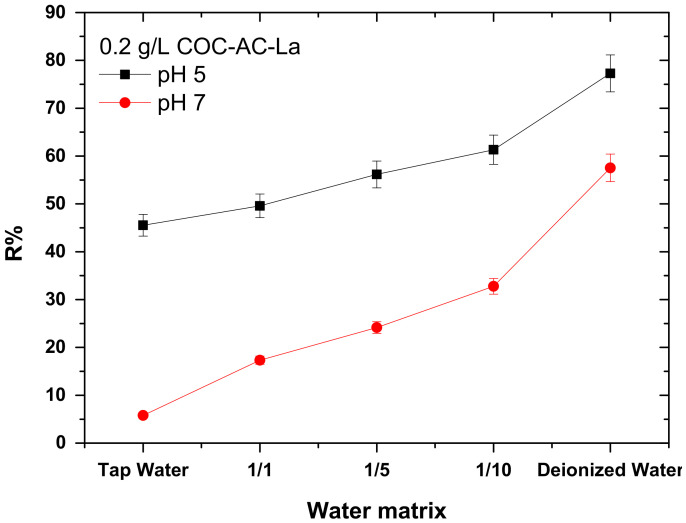
Effect of water matrix on the adsorption of Cr(VI) on COC-AC-La; initial Cr(VI) concentration 100 μg/L, solution pH 5 and 7, dose 0.2 g/L, T = 22 ± 1 °C, contact time 24 h, in deionized water.

Figure 10 presents the effect of water matrix on the adsorption of Cr(VI) on COC-AC-La in two different pH values. As exhibit, the efficiency of COC-AC-La on Cr(VI) uptake was found to increase by the dilution of tap water with distilled water, with the an improvement being better as the dilution gets 1:10 ratio, both at pH 5 and 7, thus as the concentrations of the other water components decrease.

Furthermore, by increasing the dilution of tap water, thereby reducing the concentrations of the other components of the water matrix and accordingly the conductivity and hardness, the removal of Cr(VI) was increased. This shows clearly that in tap water, the water matrix plays a crucial role, because the other water components compete with the adsorption sites with Cr(VI) and therefore, reduce the efficiency of Cr(VI) removal. However, the results indicate, that still under real conditions, this material is capable of reducing the Cr(VI) concentration to below 25 μg/L, which is the new limit of Cr in drinking water in the EU.

### 3.9. Regeneration Study

**Figure 11:**
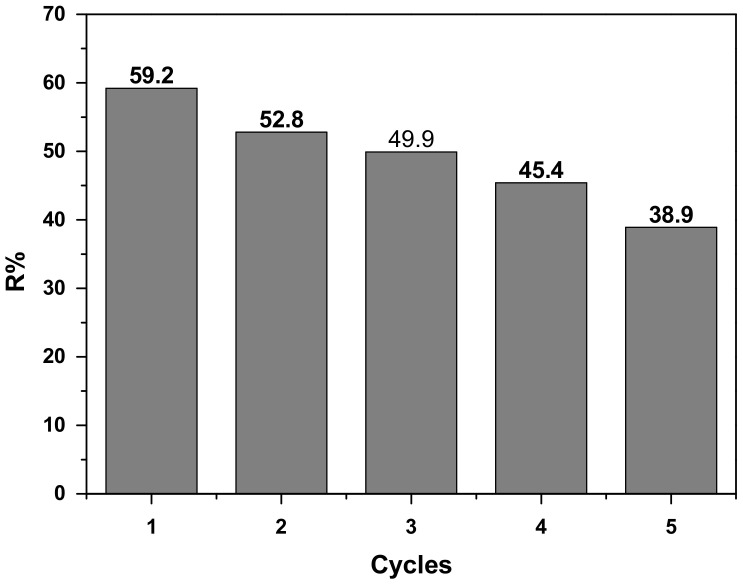
Cr(VI) adsorption on COC-AC-La; initial Cr(VI) concentration 100 μg/L, solution pH 5, dose 0.2 g/L, T = 22 ± 1 °C, contact time 2 h, for five adsorption–desorption cycles after regeneration at alkalic pH values, by using NaOH treatment.

Recycling studies were carried out in order to investigate the reusability of COC-AC-La, for the removal of Cr(VI) under similar experimental conditions for each cycle, for an initial Cr(VI) concentration of 100 μg/L and adsorbent dosage of 0.2 g/L at pH 5. After the first cycle, the used COC-AC-La particles were treated with 0.01 M NaOH [25,69,70] and shaken for approximately 2 h, then it washed with distilled water to remove the excess base amount. In order to reuse the adsorbent, it is not necessary to achieve the theoretical equilibrium time. Instead, a satisfactory repetitive time is selected in all applied cycles. As shown in Figure 11, the desorbed adsorbent was tested up to five repeated cycles for the sorption of Cr(VI). In the first cycle the percentage removal of Cr(VI) was about 59.2% and after the fifth cycle it was about 38.9%. Therefore, this study demonstrated a reuse of COC-AC-La adsorbent for five cycles for Cr(VI) removal, after successfully regenerated by using NaOH treatment.

### 3.10. Comparison with Other Materials in Literature

Table 6 compares the proposed adsorbent material of this research (COC-AC-La) with some indicative materials that appear in the recent literature, mentioning various optimal parameters. As it turns out, initially, it is confirmed that the acidic conditions are optimal (pH around 4) for the removal of Cr(VI). Where material regeneration experiments are performed, the 5 cycles are the most common, regarding the effectiveness of the adsorbent. Even if the Cr(VI) uptake on COC-AC-La is only 6.3 μg/g, it can be a good adsorbent, because comparison of adsorbent performances on the basis of adsorption capacity is not always accurate [71]. Adsorption capacity depends on various other factors including initial adsorbent concentration. Since the initial Cr(VI) concentration for most of the experiments in this study is 100 μg/L (0.1 mg/L), Cr(VI) uptake, mg/g comes to be lower. The novelty of this material (COC-AC-La) is that an activated carbon, produced by coconut shells and further modified by using lanthanum and applied to the removal of Cr(VI) from waters, is first mentioned according to recent literature. In addition, it should be noted that the dose of COC-AC-La used is very small compared to the corresponding dose used in the literature of Table 6, i.e., only 0.1 g/L to achieve a removal rate of 78%, which leads to a residual Cr(VI) concentration below the permissible limits.

## 4. Conclusions

In this study, activated carbon originating from coconut shells was modified with lanthanum chloride and used for the removal of Cr(VI) from aqueous solutions. The prepared material was characterized using SEM, FTIR, and BET techniques. Characterization results showed that the adsorbent exhibits a surface area of 139 m^2^/g with a particle size of 6.08 nm. SEM results showed that the adsorbent has a porous and homogenous structure while FTIR results, before and after adsorption, confirmed the adsorption of Cr(VI) on COC-AC-La, as indicated by the peaks of 1080 and 820 cm^−1^, most likely attributable to Cr-O-La bond.

The material COC-AC-La provided an adsorption capacity for Cr(VI) of up to 95%, depending on experimental conditions. This efficiency was much greater than that of unmodified activated carbon (44%). The removal rates of Cr(VI) increased with increased adsorbent dosage and decreased with increased pH. It was found that the maximum percentage removal was achieved at pH 5, reaching 95%, for initial Cr(VI) concentration of 30 μg/L, which was decreased to 78% for initial Cr(VI) concentration of 100 μg/L, for constant adsorbent dose of 0.2 g/L. The Freundlich isotherm model was found to better fit the adsorption, with a high correlation coefficient (R^2^ = 0.99), than the application of Langmuir isotherm model. The relative K_F_ factor was 1.2683 μg/g. The results fitted well to the pseudo-second order kinetics model with a good correlation, concluding that the adsorption of Cr(VI) on COC-AC-La was closer to chemisorption. In addition, regeneration studies were conducted using 0.01 M of NaOH and the percentage removal of Cr(VI) was about 59.2% after first cycle and about 38.9%. After the fifth cycle, after shaken for approximately 2 h, showing the adsorbent ability to be reused for several cycles.

The effect of water matrix composition on the removal of Cr(VI) was also studied in this work. Tap water originating from groundwater and dilution of it were used and showed that the efficiency of COC-AC-La for Cr(VI) uptake was found to increase as the tap water was diluted by deionized water.

## Figures and Tables

**Table 1:** Elemental and proximate analysis of waste coconut shells.

	Elemental Analysis (wt.%)
Carbon	59.42
Hydrogen	6.31
Nitrogen	0.56
Oxygen	33.63
Sulfur	0.08
	**Proximate Analysis (wt.%)**
Moisture content	9.41
Volatile constant	74.22
Fixed carbon	13.28
Ash	3.09

**Table 2:** Physical properties of COC-AC-La adsorbent.

Parameters	COC-AC-La
BET Surface area, S_BET_ (m^2^/g)	139
Micropore volume, V_micro_ (cm^3^/g)	0.035
Mesopore volume, V_meso_ (cm^3^/g)	0.144
Total pore volume, V_T_ (cm^3^/g)	1.121

**Table 3:** Constants of Freundlich isotherm model for Cr(VI) adsorption on COC-AC-La.

pH	Freundlich Isotherm Model			
	1/*n*	*n*	*K_F_* (μg/g)	R^2^
5	0.3649	2.7408	1.2683	0.9886
6	0.3091	3.2351	1.1485	0.9907
7	0.4908	2.0376	0.4368	0.9659
8	0.5623	1.7783	0.0455	0.9735

**Table 4:** Pseudo-second order Kinetics parameters for Cr(VI) adsorption on COC-AC-La.

pH	*Q_e,exp_* (μg/g)	Pseudo-Second Order Model		
		K_2_ (L/μg·min)	*Q_e,cal_* (μg/g)	R^2^
5	3.9125	0.0036	4.4954	0.9895
7	2.9123	0.0184	2.5821	0.9941

**Table 5:** Main physicochemical parameters of different water types.

Water Type		pH_init_	Conductivity (μS/cm)	Ca^2+^ (mg/L)	Mg^2+^ (mg/L)
Tap water	1 L tap water	7.3	398.5	190	30
1/1	0.5 L tap water and 0.5 L deionized water	7.2	209.3	80	15
1/5	0.2 L tap water and 0.8 L deionized water	7.2	87.3	30	5
1/10	0.1 L tap water and 0.9 L deionized water	7.2	45.4	20	2
Dist. Water	1 L deionized water	6.8	1.2	-	-

**Table 6:** Comparison of adsorption capacities and experimental conditions for Cr(VI) removal of current work with relevant literature findings.

Adsorbent	[Cr]_o_ (mg/L)	Dosage (g/L)	pH_init_	Contact Time (min)	Adsorption Capacity (mg/g)	*R%*	Recycling Cycles	Ref.
ALC	100	3.5	1.0	240	4.3	76	-	[66]
CMC-g-PAA	10	1.0	1.0	600	6.5	64	-	[67]
CS-GO	50	2.0	2.0	420	104.0	96	10	[25]
La-DEA	10	8.0	5.6	50	357.1	99	difficult to be regenerated	[43]
La-modified ceramic materials	3.0	0.5	4.0	1440	13.0	-	-	[47]
CS–La–βCD	100	2.0	4.0	30	48.4	98	5	[69]
CSFLMOH	100	2.0	4.0	60	48.3	-	5	[62]
Ce/Fe_3_O_4_	20	4.0	2.0	120	9.6	99	4	[72]
Fe-BDC@AC	25	1.0	5.5	50	79.1	61	5	[73]
AC/nZVI	10	1.5	4.0	720	6.7	63	5	[74]
SAC	10	5.0	5.0	150	2.65	73	2	[75]
Ch-ACs	10	10.0	2.0	60	20.0	99	-	[76]
COC-AC-La	0.1	0.2	5.0	240	6.3 μg/g	78	5	Present study

## Data Availability

The data presented in this study are available upon request, from the corresponding author.
